# Chloroquine treatment of ARPE-19 cells leads to lysosome dilation and intracellular lipid accumulation: possible implications of lysosomal dysfunction in macular degeneration

**DOI:** 10.1186/2045-3701-1-10

**Published:** 2011-03-08

**Authors:** Patrick M Chen, Zoë J Gombart, Jeff W Chen

**Affiliations:** 1 Department of Neurological Surgery, Legacy Clinical Research and Technology Center, 1225 NE 2nd Ave., Portland, OR 97232, USA; 2 Department of Biological Sciences, Dartmouth College, 103 Gilman Hall, Hanover, NH 03755, USA

## Abstract

**Background:**

Age-related macular degeneration (AMD) is the leading cause of vision loss in elderly people over 60. The pathogenesis is still unclear. It has been suggested that lysosomal stress may lead to drusen formation, a biomarker of AMD. In this study, ARPE-19 cells were treated with chloroquine to inhibit lysosomal function.

**Results:**

Chloroquine-treated ARPE-19 cells demonstrate a marked increase in vacuolation and dense intracellular debris. These are identified as chloroquine-dilated lysosomes and lipid bodies with LAMP-2 and LipidTOX co-localization, respectively. Dilation is an indicator of lysosomal dysfunction. Chloroquine disrupts uptake of exogenously applied rhodamine-labeled dextran by these cells. This suggests a disruption in the phagocytic pathway. The increase in LAMP protein levels, as assessed by Western blots, suggests the possible involvement in autophagy. Oxidative stress with H_2_O_2 _does not induce vacuolation or lipid accumulation.

**Conclusion:**

These findings suggest a possible role for lysosomes in AMD. Chloroquine treatment of RPE cells may provide insights into the cellular mechanisms underlying AMD.

## Background

Age-Related Macular Degeneration (AMD) is the leading cause of progressive central vision loss in elderly people over the age of 60 [1-3]. The clinical hallmarks of "dry" AMD, which accounts for 85-90% of AMD patients, is the appearance of yellow pigments known as drusen and marked photoreceptor death within the macula [1,4]. While it has been established that smoking, light exposure and genetics are risk factors for AMD, its cellular-molecular pathogenesis remains unclear [4].

Retinal pigment epithelium (RPE) metabolism is an important factor in drusen buildup along the Bruch's membrane, located strategically between the choroid and RPE [4]. The RPE, a highly specialized monolayer epithelium that forms the outermost layer of the retina, is among the most active phagocytic systems in the body [5,6]. On a daily basis, the outer segment tips of photoreceptors are phagocytosed into the RPE, and digested in phago-lysosomes within the RPE [7]. Autophagy also contributes to the heavy load of material the RPE digests [8]. In theory, lysosomal overload may thus lead to a buildup of biological "waste products", reducing RPE efficiency and contributing to extracellular protein-lipid deposits along Bruch's membrane [4,8-10].

Lysosomal overload and dysfunction in RPE is suspected to be a critical and early cause of AMD [4,11]. It is well established that lipofuscin, a pigmented aggregate of proteins and lipids, a primary component of drusen, and an AMD biomarker, is sequestered by lysosomes in RPE [12,13]. At critical concentrations, N-retinylidene-N- retinylethanolamine (A2-E), a fluorescent pigment of lipofuscin, inhibits lysosomal ATPase proton pumps, inhibits critical enzymes and causes lysosomal compartment leakage into RPE cytoplasm [4,14,15]. Recently, it has been shown that the variant B mutation in cystatin C, a widely expressed lysosomal protease inhibitor, inhibits proteolytic regulator secretion, mistargets signaling, causes inappropriate cell protein retention and is associated with AMD and Alzheimer's [16]. Furthermore, proteins modified by lipid peroxidation similar to those found in lipofuscin have been shown to reduce proteolytic activity of lysosomes of RPE cells [17]. Finally, studies have begun to uncover a link between retinal degeneration and Niemann-Pick type C, a known lysosomal storage disease. A recent study observed that mice with mutations in the Npc1 and Npc2 gene, which transcribe proteins that mediate the exit of lipoproteins from lysosomes, demonstrate striking retinal degeneration, upregulation of autophagy and marked lipofuscin accumulation within the RPE [18]. These aforementioned studies suggest that abnormalities in the structural integrity and enzymatic activity of the lysosomes of RPE cells may play a role in the pathogenesis of AMD.

In this study, we investigate the possible role of lysosomes in AMD by treating *in vitro *human adult retinal pigmented epithelium-19 (ARPE-19) cells, which have previously been used as a model for the study of the etiology and development of AMD [19,20], with chloroquine, a known lysosomotropic agent. The effects of chloroquine as a retinopathic agent, as observed by lysosomal dysfunction and RPE degradation, have been demonstrated in various animal models [21-24]. We use the ability of chloroquine to increase pH [25] to both understand the general effects of chloroquine on ARPE-19, and as a model for lysosomal inhibition. The results demonstrate that chloroquine induces vacuole formation, cell death, cytosolic lipid buildup and decreased exogenous dextran uptake in ARPE-19.

## Results

### ARPE-19 Lysosomal Inhibition with Chloroquine Treatment

Chloroquine is a known lysosomotropic agent that increases lysosomal pH by accumulating within lysosomes as a deprotonated weak base. To study the effects of lysosomal dysfunction in ARPE-19, it was necessary to establish an *in vitro *model utilizing chloroquine. We determined the concentration of chloroquine that substantially changed lysosomal activity, but did not result in cell necrosis.

**Figure 1:**
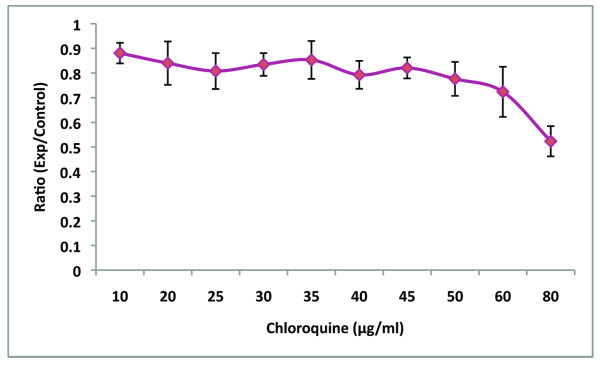
**Cell Viability Assay**. MTT assay shows chloroquine toxicity is both time and dose dependent. Chloroquine concentrations of 10-30 μg/ml (p < 0.05) do not significantly affect cell viability.

To find an optimal concentration of chloroquine that did not affect ARPE-19 cell viability, we utilized both DAPI nuclei staining and the MTT assay (Figure 1). For DAPI cell quantification, the nuclei of ten random areas of the coverslip were counted. The results (not shown) of these counts were averaged, expressed as the percentage of the control, and analyzed by student t-test and one-way ANOVA with a TI-89 Texas Instruments graphing calculator. The DAPI analysis showed cell viability was time and dosage dependent, with concentrations of 10-20 μg/ml at 24 hour incubation periods not significantly (p < 0.05, n = 6) affected.

The MTT assay, which evaluated cell proliferation by measuring metabolic activity, showed similar results. Figure 1 demonstrates that cell viability and metabolism is relatively unaffected from 10-30 μg/ml and is dosage dependent. Student t-test shows no significant difference from 10-20 μg/ml (p < 0.05, n = 6) with significant difference in cell viability between 10 and 40 μg/ml (p = 0.033, <0.05, n = 6).

### Chloroquine Induces Cytosolic Vacuolation and Dense Body Formation

**Figure 2:**
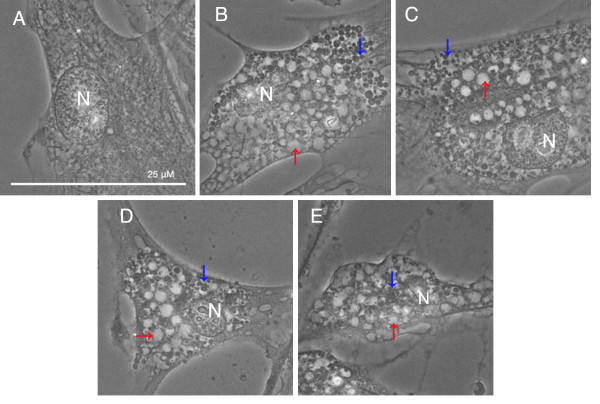
**Cytoplasmic Changes with Chloroquine in ARPE-19**. Phase contrast microscopy of ARPE-19 with 0 (A), 10 (B), 20 (C), 40 (D), 80 (E) μg/ml chloroquine. Chloroquine treatment causes membrane-enclosed vacuoles (red arrows) and membranous dense bodies (blue arrows). ARPE treated with high chloroquine dosage show signs of cell death. Bar size = 25 microns.

**Figure 3:**
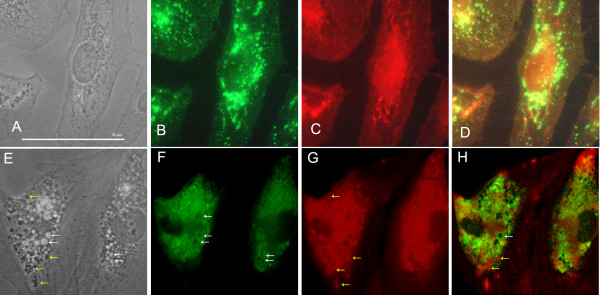
**Co-localization of LAMP-2 and LipidTOX**. (A-D) Control ARPE-19. (E-H) 20 μg/ml chloroquine-treated ARPE-19. Control phase contrast (A) shows no vacuolation and LAMP-2 (B, green), and LipidTOX(C, red) staining is observed throughout cell. D is fluorescent overlay. Chloroquine-treated cell in phase contrast (E) shows intense vacuolation (white arrows). These vacuoles co-localize with LAMP-2 staining (F, corresponding white arrows). Chloroquine treatment also induces dense body formation (E, yellow arrows). Dense formations co-localize with neutral lipid (G, corresponding yellow arrows). Overlay (H) shows no overlap between lipids and vacuoles. Bar size = 25 microns.

**Figure 4:**
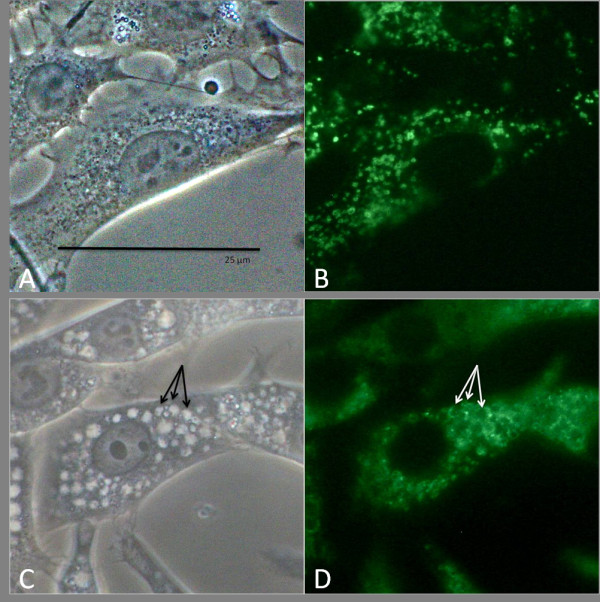
**Co-localization of LAMP-1 in NIH/3T3**. NIH/3T3 cells show a similar pattern of vacuole formation with treatment of chloroquine. Phase contrast and corresponding LAMP-1 co-localization of 25 μg/ml chloroquine-treated cells (C, D) show vacuolation of LAMP-1, when compared with control (A, B). The arrows in C and D note that the borders of the vacuoles correspond with LAMP-1 protein. Bar size = 25 microns.

Having determined that low concentrations (10-20 μg/ml) at 24-hour incubation did not significantly reduce viability, we evaluated the cytoplasmic cellular changes in ARPE-19 with phase contrast microscopy. Figure 2 shows the progression of cytoplasmic changes of ARPE-19 treated with chloroquine under phase contrast microscopy. We found that 10-20 μg/ml of chloroquine induced marked vacuolation within the cytoplasm of ARPE-19, as can be seen in Figure 3. In addition to increased vacuole size and dispersion, we note the appearance of dense, black, circular formations interspersed throughout the cytoplasm and around the large vacuoles. Vacuole and dense body size increased with chloroquine dosage. In a parallel study performed on NIH 3T3 cells, we noted the dilation of lysosomes at similar concentrations of chloroquine treatment (Figure 4). However, there was no concurrent development of the dense, black formations.

### Lamp-2 Co-localizes with Dilated Vacuoles, LipidTOX Co-localizes with Dense Formations

To determine if the abnormal vacuoles were dilated lysosomes, as documented in a previous study using calf RPE [26], and if the dense bodies contained lipid, ARPE-19 cells were doubled stained with LAMP-2 antibody and LipidTOX neutral lipid. Inhibition of the essential lysosomal protease cathepsin B and other mucopolysaccharides is characteristic of chloroquine-dilated lysosomes [25]. Moreover, lipid co-localization would suggest a form of intracellular "biological debris" accumulation within the cell in response to lysosomal enzymatic inhibition.

**Figure 5:**
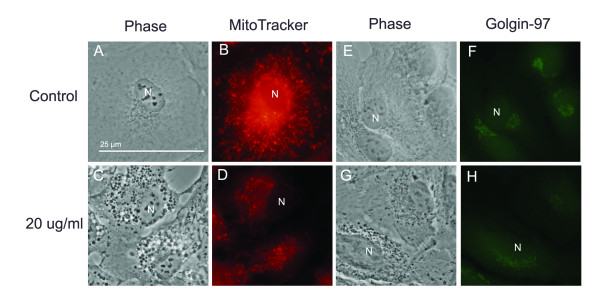
**Chloroquine Effect on ARPE-19 Golgi and Mitochondria**. (A-D) Mitochondria co-localization in (A-B) control and (C-D) 20 μg/ml-treated ARPE-19. There is no difference in MitoTracker staining (red) intensity, shape or localization between control (B) and treatment (D). Similarly, there were no noticeable changes in Golgi apparatus (E-H). Golgi co-localization of control (E-F) and 20 μg/ml chloroquine treatments. Golgi is stained with Golgin-97 antibody staining (green). Bar size = 25 microns.

LAMP-2 antibody staining of chloroquine-treated cells was used to co-localize dilated vacuoles with lysosomes and phago-lysosomal complexes. As shown in Figure 3 LAMP-2 antibody staining co-localizes with membranes of dilated vacuoles. We did not observe noticeable difference in the strength of antibody staining between control and chloroquine-treated cells. LipidTOX staining localized in the perinuclear and peripheral area of the cell. Figure 3 shows co-localization of dense bodies with LipidTOX, especially in the periphery of cells. As a control for non-specific staining, cells washed with Triton × showed no LipidTOX staining. Chloroquine caused no morphological changes in the Golgi apparatus or mitochondria (Figure 5). Figure 5 shows no co-localization, and there was no marked decrease in mitochondrial staining between control and 10-20 μg/ml chloroquine treatment. It appears that co-localized lipids may represent intracellular debris, the consequence of the dysfunctional lysosomes.

### ARPE-19 Phagocytic Pathway is Disrupted by Chloroquine

**Figure 6:**
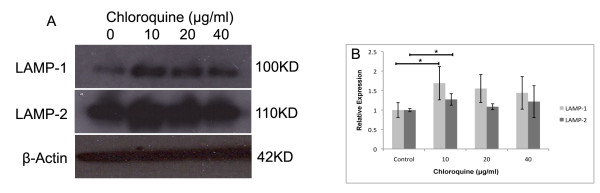
**Phagocytic Activity and LAMP Protein**. (A) Comparisons of LAMP-1 and LAMP-2 protein levels at different concentrations of chloroquine. Protein levels measured in ARPE-19 by Western blot with beta-actin as loading control. Western shows marked qualitative increase in LAMP 1-2 band size between control and 10 μg/ml chloroquine treatment, while loaded control is constant. (B) There is a significant increase in LAMP-1 and 2 (p < 0.05) at 10 μg/ml with overall upregulation trend response to chloroquine treatment. Immunoblots (n = 6) were scanned, and densitometry was performed on bands with ImageJ (NIH, Bethesda MD). Relative band density measurement was repeated several times to ensure maximum and minimum values.

We use Western blotting of LAMP-1 and 2 (Figure 6A) and exogenous rhodamine-labeled dextran (Figure 6B) to observe disruption of chloroquine-treated autophagic and phagocytic pathways, respectively. If phagocytic function is normal, LAMP-1 and LAMP-2 protein levels should remain relatively the same despite chloroquine treatment. Quantitative examination of the Western blots was performed by densitometry [27] (ImageJ Software, available at http://rsb.info.nih.gov/ij/). Western blots in Figure 6 show marked increase of LAMP-1 in chloroquine-treated cells, especially at 10 μg/ml. LAMP-2 shows a similar, but less drastic, upregulation trend.

**Figure 7:**
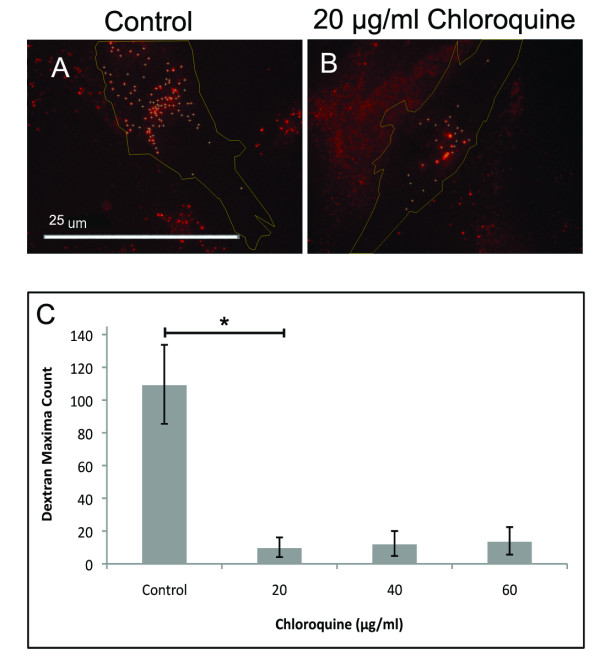
**Phagocytic Activity Measured by Dextran Uptake**. (A-B) Immunofluorescence of exogenous rhodamine-labeled dextran (red) uptake with ImageJ markings (crosshairs) of counted dextran maximas. 20 μg/ml chloroquine-treated cells at 24 hours (B) showed striking decrease in fluorescent dextran uptake when compared to control (A). (C) Quantization of dextran uptake between control and chloroquine-treated ARPE-19. There is a significant (p < 0.05) decrease in relative dextran uptake in individual sampled ARPE-19 cells between control and chloroquine-treated cells. Quantitation was performed by using ImageJ, isolating cells of relative same size (~20-25 microns), and then calculating florescent maximas (tolerance = 20, based on control). Maximas were averaged and standard t-test was performed (n = 10).

Exogenous rhodamine-labeled dextran was applied to the chloroquine-treated cells. We observe a striking decrease in intracellular dextran in chloroquine-treated cells (Figure 7A). Dextran retention and uptake was quantified by comparing relative maximas with ImageJ. There is a significant decrease in intracellular dextran between the control and 10 μg/ml treatment (Figure 7B). These findings suggest either increased exocytosis or decreased phagocytic uptake.

### H_2_O_2 _Oxidative Stress Test

**Figure 8:**
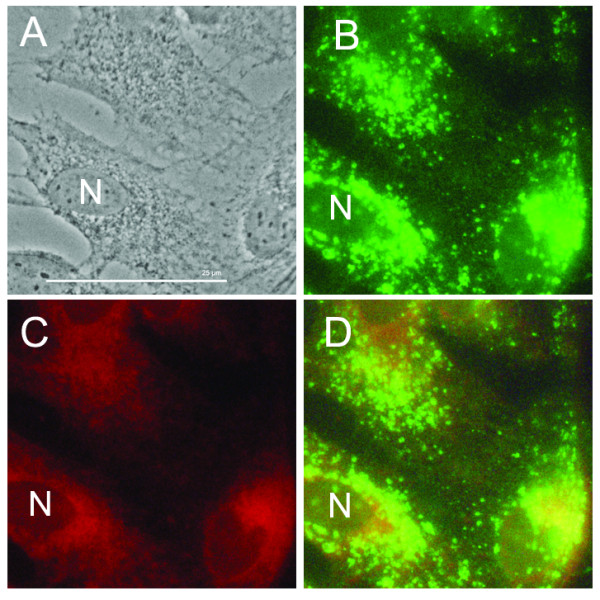
**Oxidative Stress Test**. (A) phase contrast, (B) LAMP-2 and (C) LipidTOX staining of 10 mM hydrogen peroxide-treated ARPE-19. (D) Overlay showing co-localization of neutral lipid and LAMP-2. There are no differences in cell cytoplasm (vacuoles, lipids) in phase or fluorescence between control (Fig 2 A-D) and hydrogen peroxide treatment.

Oxidative stress is one of the primary causes of AMD, and is suspected as a primary catalyst of the disease [28]. We investigated whether intracellular lipid accumulation occurs to the same extent in ARPE cells treated with H_2_O_2_. No cytoplasmic dense aggregate formation or lysosomal dilation occurred in H_2_O_2_-treated cells under phase contrast (Figure 8). There were no striking differences in LAMP-2 and LipidTOX co-localization staining patterns in control and H_2_O_2_-treated ARPE-19 cells. This suggests that aforementioned lipid buildup in ARPE-19 observed with chloroquine treatment is accelerated by induced alteration in lysosomal function, and not secondary to oxidative stress.

## Discussion

The molecular mechanisms by which drusen and AMD develop are not clearly understood at this time. Drusen has been postulated to be the combination of oxidative stress, lipofuscin and POS overload lysosomal capacity [4]. Here, we investigate the possible role of lysosomal malfunction in AMD by examining the effects of chloroquine, a lysosomotropic agent, on ARPE-19 cells. Our findings detail a time-dosage toxicity curve, marked vacuolation in the cytoplasm, intracellular debris accumulation, and altered phagocytic pathways in chloroquine treated ARPE-19.

Chloroquine proved to be an apt *in vitro *model for observing the buildup of intracellular lipid in ARPE-19 cells. Chloroquine induced intense vacuolation of cells. A similar observation has recently been observed under similar conditions [29] and in bovine RPE under slightly hypoxic conditions [23]. Vacuolation has been shown as an indicator of altered acidification through a proton "trapping" mechanism [25] and inhibition of key lysosomal enzymes, including cathepsin D [30]. This is consistent with *in vivo *findings that have demonstrated that retinal aging has been associated with a decrease in lysosomal glycosidases and downregulation of cathepsin D [31]. We further confirm the validity of dilation, and thus induced inhibition of phago-lysosomes, by successful co-localization with LAMP-2. Perhaps the most striking finding was the co-localization of dense formation buildup with lipid staining. The lack of dense bodies in 3T3 cells (Figure 4) suggests that this phenomenon may be unique to cells of the retinal epithelium. RPE cells by the nature of their function to phagocytose POS are likely to be more dependent upon lysosomal activity. Aberrations in this activity may lead to the accumulation of the lipid bodies that ultimately may contribute to drusen formation. Finally, in contrast to the well-established mitochondrial-axis theory [32], we found that the accumulation of lipid, induced by chloroquine, is not seen with an oxidative stress model (Figure 8), which is possibly only a secondary factor.

Interestingly, increased LAMP-1 and 2 protein levels, as well as decreased retention/uptake of exogenous dextran, suggest that alterations in the phagocytic pathway occur after lysosomal inhibition by chloroquine. Upregulation of LAMP could indicate a number of intracellular changes. It could be a reflection of a general increase in lysosomal surface membrane size due to dilation, or an overall increase in lysosomes. Furthermore, it may indicate a compensatory upregulation in phagosome-lysosomal fusion and movement [33]. It is possible that the striking increase in LAMP-1 is an indication of the onset of apoptosis, an observation noted in glioblastoma cells in culture [34]. In contrast, chloroquine at low dosages does not seem to induce increased autophagy, as autophagic-associated vacuolation is correlated with decreased LAMP-2 levels [35]. At the time of publication, we have initiated studies on siRNA knockdown of LAMP protein to eludicate the mechanism of these protein changes in light of vacuolation.

Our exogenous dextran findings suggest either a lack of intracellular retention or increased exocytosis due to lysosomal inhibition. There is much debate on the mechanisms that occurs in AMD. There is a belief that the combination of oxidative stress and a decrease in ATP drives the exocytosis of partially digested intracellular proteins into the Bruch's membrane [8]. However, recent studies have investigated the role of ABCA4 transporter mutations in reducing movement of oxidized lipids in Stargardt's AMD [36]. Similarly, it has been shown that oxidized lipid-proteins can block phagosome formation, and thus deter internalization into RPE [37].

**Figure 9:**
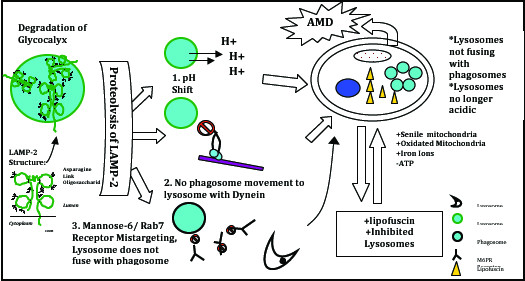
**Possible LAMP-2/Lysosomal Inhibition Model of Pathogenesis of AMD**. An unknown cause results in loss of the protective glycocalyx of LAMP-2. Proteolysis of LAMP-2 occurs. Loss of LAMP-2 results in either 1) A pH shift and loss of acidity of lysosome, 2) dynein no longer moving late phagosome to microtubule sorting center near Golgi for fusion with lysosome or 3) perturbation of M6PR/Rab recycling such that M6PR/Rab7 does not tag late endosomes. The loss of functionality of M6PR/Rab7 results in a lack of phagosome lysosome-fusion. Any of these results in loss of the lysosome's ability to degrade intra- and extracellular material. Subsequently, undegraded material is oxidized, turning to lipofuscin. Ultimately, the combination of oxidative stress, Fe+ accumulation, senile mitochondria and decrease in ATP results in inefficient turnover of organelles and increased inhibition of lysosomes. RPE death triggers photoreceptor and macula death.

Another candidate for the cause of lysosomal dysfunction in cells is the degradation of the glycocalyx, which protects the vital LAMP-2 membrane protein. While the exact role of LAMP-2 is still unclear, it has been proposed that it is responsible for equilibrating proton levels in lysosomes. Moreover, recent studies suggest LAMP-2 may be critical in communication between phagosomes and lysosomes, and in receptor-targeting for lysosome maturation [38-40]. We therefore postulate a "LAMP-2/Lysosomal Inhibition" AMD biogenesis model (Figure 9). In this postulated model, lysosomal inhibition begins with the modification or degradation of the asparagine-linked glycocalyx of LAMP-2. Removal of the glycocalyx results in rapid proteolysis of LAMP-2 [41]. The glycocalyx shield could be slowly modified or degraded over time by the powerful proteases within the lysosomal lumen, perhaps as a result of aging. Further investigation will be required to understand the causes of the breakdown of the glycocalyx. Nevertheless, after LAMP-2 has been degraded, a pH shift occurs, dynein is no longer able to pull mature phagosomes to lysosomes or mistargeting of M6PR/Rab7 occurs [42]. Consequently, the lysosome is incapable of degrading extra- and intracellular material by phagolysosomal fusion. This results in the accumulation of oxidized lipofuscin. The combination of mitochondrial oxidative stress and amassing of senile, inefficient mitochondria and iron in lysosomes further expedites lipofuscin buildup.

In summary, these studies demonstrate two new findings. First, we show the morphological changes in ARPE-19 due to chloroquine. Also, we demonstrate that lysosomal dysfunction leads to the accumulation of lipids and their byproducts in ARPE-19 cells which may simulate the situation in AMD. These findings seem to be specific for this particular cell line, and we demonstrate that despite lysosome dilation with chloroquine treatment, this profuse lipid accumulation is not seen with NIH 3T3 cells (Figure 4), neuroblastoma or glioblastoma cell lines that we have tested. This is perhaps a reflection of the intense lysosomal/autophagocytic activity of the RPE cells. These cells may be more susceptible to perturbations of the lysosomal degradation systems and are thus a good model for studying potential mechanisms of AMD. There is a piqued interest in the relationship between lysosomal inhibition and AMD as seen in the recent literature on the correlation between iron overload and reduced Cat-D activity [43], the conversion of protease substrates to lysosomal enzyme substrate inhibitors [44] and the possible usage of toxins to elevate low pH in ARPE lysosomes [45].

## Conclusions

These studies demonstrate the dose and time dependent response of ARPE-19 to chloroquine. Moreover, we have demonstrated that chloroquine induces intracellular vacuolation and lipid accumulation, and disrupts the phagocytic pathway. Our observations appear to be independent of oxidative stress. Lysosomal malfunction may thus have a pivotal role in the pathogenesis of AMD.

## Methods

### Cell Culture Growth Technique

Human ARPE-19 tissue culture cells were obtained from American Tissue Culture Center, Rockville, MD (ATCC, CRL-2502). Cells were grown in Ham's F12 Media: DMEM (1:1) (ATCC, 30-2006) with 10% fetal bovine serum (FBS) (ATCC, 30-2030) as monolayers at 37°C with 5% CO_2 _in 100 × 20 mm round culture dishes (Falcon, 35-3003). NIH/3T3 cells, embryonic mouse fibroblast cells, were also obtained from ATCC (CRL-1658) and grown in DMEM (ATCC, 30-2002) with 10% calf bovine serum (ATCC, 30-2030) at the same conditions as ARPE cells. The cells were propagated by splitting at a ratio of 1:5 with a trypsin-EDTA solution. Studies were done on both ARPE-19 and 3T3 cells during passages 5-15.

### Chloroquine

Chloroquine was obtained from Sigma (C6628, St. Louis, MO). Chloroquine stock solutions were made at 10 mg/ml in double deionized water, and sterilized by filtration with an Acrodisc syringe filter with a 0.2 μm Tuffryn membrane (Pall Life Sciences, New York). Aliquots of the sterile stock solutions were stored at -20°C until use. The appropriate amounts of stock solution were added to the media to achieve the desired concentration. To test the effect of the carrier, equivalent volumes of sterile water were also added to a set of ARPE-19 cells.

### Response of ARPE-19 Cells to Chloroquine

Approximately 50,000 cells were seeded into each well of a 12-well plate (Falcon 35-3043) and grown to 50% confluency on sterile glass coverslips. These were incubated with chloroquine at concentrations of 0, 10, 20, 40, 80 and 100 μg/ml for 24 hours. In a separate set of experiments, cells were incubated at 0, 40 and 80 μg/ml for 2 hours and 6 hours. To demonstrate that the carrier did not affect the ARPE-19 cells, sterile water, in a quantity equivalent to that of the carrier, was added to a triplicate set of RPE cells. Each point on the graph in Figure 1 represents seven independent experiments. The loss of cells after the different chloroquine treatments was determined by counting the cells on the coverslip. To visualize the cells, 4'-6-Diamidino-2-phenylindol (DAPI) was used to stain the cell nuclei. After the designated treatment, coverslips were fixed *in situ *in 3.7% paraformaldehyde. Coverslips were washed three times with 1X phosphate buffered saline (PBS), and then once with PBS containing 1 μg/ml DAPI and 0.1% saponin. The stained cells were visualized through a DAPI filter with a 20× panfluorochrome objective with a BX50 Olympus microscope. Ten random fields from each coverslip were photographed and counted.

### Cell Proliferation Assay

An MTT assay kit was obtained from Roche (11465007001). Approximately 5,000 cells were seeded into each well of a 96-well plate, and were grown to ~50% confluency. Chloroquine treatments of 20, 30, 40, 50, 60, and 80 μg/ml were administered in 100 μl of media/well, and cells were incubated for 24 hours at 37°C. After this first incubation period, 10 μl of MTT labeling reagent was added to each well, bringing the final concentration of MTT labeling reagent to 10 mg/ml. The cells were incubated for 4 hours. At this point, 100 μl of the solubilization solution was added to each well, and the plate was incubated overnight. After this last incubation period, the spectrophotometric absorbance of the samples was measured using an ELISA reader.

### LAMP-2 and Cy2 Secondary Antibodies

Anti-human LAMP-2 monoclonal antibody (anti-mouse IgG_1_) was obtained as stock hybridoma tissue supernatants from The Developmental Studies Hybridoma Bank (University of Iowa) and diluted (1:50) in a 0.1% Bovine Albumen Serum (BSA)/0.1% saponin/PBS solution. The secondary goat-anti-mouse cy2 or rhodamine antibody (Kirkegaard and Perry, Gaithersburg, MD) was diluted (1:60) with 0.1% BSA/0.1% saponin/PBS.

### LAMP-2 Immunofluorescent Staining

RPE and 3T3 cells were stained for LAMP-2 as previously described [46].

### Neutral Lipid Stain

Stock HCS LipidTOX Red Neutral Lipid Stain (Invitrogen, H34475) was diluted 1:4000 in PBS. Coverslips with LipidTOX were incubated for 15-20 minutes at room temperature. LipidTOX was then removed and cells were washed once with 1X PBS. To test LipidTOX specificity, cells were washed with 0.1% Triton X-100 in PBS prior to staining with LipidTOX. Triton X-100-washed cells functioned as a control, demonstrating no staining with the LipidTOX.

### Epifluorescence and Phase Contrast Microscopy

An Olympus BX50 microscope with phase contrast and epifluorescent capabilities, attached to an Olympus DP11 or DP20 digital camera, was used for DAPI (20×) images. This was also used for phase contrast and fluorescent-labeled studies (60×, oil immersion).

### Mitochondria Staining

MitoTracker CMX Ros Red (Invitrogen, M7512) was dissolved in dimethyl sulphoxide (DMSO [Sigma]) to achieve a final stock concentration of 1 mM. Cells were grown to 50% confluency and treated with chloroquine concentrations of 10, 20 and 40μg/ml. To stain the mitochondria, cells were incubated for 15 minutes at 37°C in fresh media with MitoTracker at a concentration of 300 nM, and then fixed with 3.7% paraformaldehyde. Cells were washed in 1× PBS three times, viewed with epifluorescence, and photographed as previously described.

### Golgi Stain

Anti-Golgin 97 mouse IgG_1 _(Invitrogen, 49363A) was dissolved in 1× PBS to create a final concentration of 200 μg/ml. The cells were incubated with anti-golgin 97 and the LAMP-2 antibodies, both diluted 1:50 in 0.1% BSA/0.1% saponin/PBS. The Golgi stain was visualized with rhodamine-labeled goat anti-mouse secondary antibody (Kirkegaard and Perry, Gaithersburg, MD).

### Western Blotting

Western blots were performed with modifications for LAMP-2 [39]. LAMP-2 was administered at a concentration of 1:2000, followed by incubation with goat anti-mouse peroxidase-conjugated secondary antibodies (BioRad, 1:3000). Antigen-antibody complexes were detected using chemiluminescence reagent kit (Perkin-Elmer ECL). The blots were stripped and then probed with actin to function as a control.

### H_2_0_2 _Oxidative Stress Study

A stock concentration of 3% hydrogen peroxide (H_2_O_2_, 0.88 M) was diluted in the growth media to attain final concentrations ranging from 0.2 to 10 mM [28]. The treated cells were incubated for 24 hours. Following incubation, cells were visualized by phase contrast microscopy and stained with LAMP-2, LipidTOX, Anti-Golgin 97, and MitoTracker.

### Dextran Uptake Studies

Rhodamine-conjugated dextran, avg. 10,000 MW, was obtained from Invitrogen. A stock solution of dextran diluted in sterile, deionized water was prepared. Cells were grown in different concentrations of chloroquine on round glass coverslips in 24-well plates for 24 hours. After 24 hours of incubation, the media was removed and replaced with new media containing the same amount of chloroquine, as well as an additional 0.05 mg/ml of rhodamine-conjugated dextran. At this point, the cells were incubated for 6 hours, and then fixed in 3.7% paraformaldehyde in PBS. Cells were mounted in a 10% glycerol-PBS solution, visualized, and photographed immediately.

## Competing interests

The authors declare that they have no competing interests.

## Authors' contributions

PC and JC conceived, designed and carried out these experiments. PC performed analysis and drafted manuscript. ZG carried out experiments and performed statistical analysis. All authors read and approved the final manuscript.
